# Predictors of a high incidence of opportunistic infections among HIV-infected children receiving antiretroviral therapy at Amhara regional state comprehensive specialized hospitals, Ethiopia: A multicenter institution-based retrospective follow-up study

**DOI:** 10.3389/fped.2023.1107321

**Published:** 2023-05-02

**Authors:** Gebrehiwot Berie Mekonnen, Binyam Minuye Birhane, Melaku Tadege Engdaw, Wotetenesh Kindie, Amare Demsie Ayele, Amare Wondim

**Affiliations:** ^1^ Department of Pediatrics and Child Health Nursing, College of Health Sciences, Debre Tabor University, Debre Tabor, Ethiopia; ^2^ School of Public Health, University of Technology Sydney, Sydney, NSW, Australia; ^3^ Social and Population Health Unit, College of Health Sciences, Debre Tabor University, Debre Tabor, Ethiopia; ^4^ Department of Surgical Nursing, School of Nursing, College of Medicine and Health Sciences, and Specialized Hospital, University of Gondar, Gondar, Ethiopia; ^5^ Department of Pediatrics and Child Health Nursing, School of Nursing, College of Medicine and Health Sciences, Specialized Hospital, University of Gondar, Gondar, Ethiopia

**Keywords:** antiretroviral therapy, HIV-infected, children, opportunistic infection, predictors, CD4

## Abstract

**Introduction:**

Globally, opportunistic infections are the leading causes of morbidity and mortality among HIV-infected children, contributing to more than 90% of HIV-related deaths. In 2014, Ethiopia launched and began to implement a “test and treat” strategy aiming to reduce the burden of opportunistic infections. Despite this intervention, opportunistic infections continue to be a serious public health issue, with limited evidence available on their overall incidence among HIV-infected children in the study area.

**Objective:**

The study aimed to assess the incidence of opportunistic infections and to identify predictors of their occurrence among HIV-infected children receiving antiretroviral therapy at Amhara Regional State Comprehensive Specialized Hospitals in 2022.

**Methods:**

A multicenter, institution-based retrospective follow-up study was conducted among 472 HIV-infected children receiving antiretroviral therapy at Amhara Regional State Comprehensive Specialized Hospitals from May 17 to June 15, 2022. Children receiving antiretroviral therapy were selected using a simple random sampling technique. Data were collected using national antiretroviral intake and follow-up forms *via* the KoBo Toolbox. STATA 16 was used for data analyses, and the Kaplan–Meier method was used to estimate probabilities of opportunistic infection-free survival. Both bi-variable and multivariable Cox proportional hazard models were employed to identify significant predictors. A *P*-value <0.05 was taken to indicate statistical significance.

**Results:**

Medical records from a total of 452 children (representing a completeness rate of 95.8%) were included and analyzed in the study. The overall incidence of opportunistic infections among children receiving ART was 8.64 per 100 person-years of observation. The predictors of elevated incidence of opportunistic infections were: a CD4 cell count below a specified threshold [AHR: 2.34 (95% CI: 1.45, 3.76)]; co-morbidity of anemia [AHR: 1.68 (95% CI: 1.06, 2.67)]; ever having exhibited only fair or poor adherence to ART drugs [AHR: 2.31 (95% CI: 1.47, 3.63)]; never having taken tuberculosis-preventive therapy [AHR: 1.95 (95% CI: 1.27, 2.99)]; and not having initiated antiretroviral therapy within 7 days of HIV diagnosis [AHR: 1.82 (95% CI: 1.12, 2.96)].

**Conclusion:**

In this study, the incidence of opportunistic infections was high. Early initiation antiretroviral therapy has direct effect on boosting the immunity, suppressing viral replications and increases the CD4 count, so that the occurrence of opportunistic infection will reduce the incidence of OIs.

## Background

Opportunistic infections (OIs) occur more frequently and with higher severity among individuals with low immunity, such as people living with human immunodeficiency virus (PLHIV) (1, 2). Immunosuppression is the hallmark of HIV infection; this predisposes PLHIV to OIs and contributes greatly to HIV-related morbidity and mortality in HIV-infected children across the globe (3). Opportunistic infections mainly affect the neurological, gastrointestinal, respiratory, and integumentary systems (4, 5).

As per the 2020 statistical report on global HIV and acquired immune deficiency syndrome (AIDS), among 37.7 million PLHIV, 1.7 million were children (6); and, according to the 2019 report, nearly 95,000 HIV-infected children died, mainly due to OIs (7).

The incidence of HIV-related OIs remains high in developed and resource-limited countries, as illustrated by the incidence by person-year observation (PYO) in the United States of America (4.99), Latin America (1.1 and 23.5), Brazil (2.63), and Ethiopia (5.53 and 9.7) (8–13). Globally, OIs are the leading cause of HIV-associated morbidity and mortality, accounting for more than 90% of all HIV-related fatalities (5, 10, 14). In developed countries, the life expectancy of newly HIV-infected children receiving antiretroviral therapy (ART) has reached the lifespan of the general population (15), in contrast, sub-Saharan African children have shorter life expectancies due to the high burden of OIs (16, 17). Among all HIV-related OIs, pneumonia and tuberculosis (TB) are the leading causes of hospital admissions for HIV-infected children receiving ART in Europe and East China, and across low- and middle-income countries (4, 8, 12, 14, 18).

Existing studies conducted in the USA (5, 19), Brazil (20), India (21), Nigeria (22), Uganda (23), South Africa (24), Tanzania (25), and Ethiopia (8, 12, 26–29) have identified baseline cluster of differentiation 4 (CD4) cell counts, poor and fair ART adherence, not taking cotrimoxazole preventive therapy (CPT), TB preventive therapy (TPT), and co-morbidity of malnutrition as contributing factors for the occurrence of OIs, even in the ART era.

In developing countries, the increased rate of OIs among HIV-infected children is related to inadequate medical care; challenges in ART adherence; delays in the provision of effective HIV treatment (5); less advanced technology for early diagnosis, prevention, and management of OIs; and lack of prophylaxis (26). Unless OIs are diagnosed and treated early, they markedly affect the treatment outcomes of PLHIV, which leads to poor quality of life, hastening of disease progression, and increasing medical costs, creating the potential risk of treatment failure and impairing the patient's response to ART regimens (30, 31).

In response to this problem, Ethiopia has implemented the Health Sector Transformation Plan II (HSTP-II), with a target of ensuring that 95% of HIV patients are receiving ART in order to achieve effective viral suppression, which in turn should result in a reduction of the incidence of OIs by the year 2025 (32).

Similarly, the Federal Ministry of Health in Ethiopia sets strategies to reduce the occurrence of OIs through reduction of HIV exposure, preventive therapies, immunization (4), and the use of highly active antiretroviral therapy (HAART). Many initiatives have already been adopted to reduce the incidence of OIs based on the suggested solutions. However, OIs remain the main factor in morbidity and mortality of children with HIV. In the meantime, Ethiopia has also adopted a “test and treat” strategy that has been in operation since June 10, 2014, targeting initiation of ART for all children who test positive for HIV, regardless of their CD4 cell count or WHO clinical stage; the aim of this initiative is to reduce HIV-related mortality and morbidity due to OIs (33, 34).

Despite the implementation of the test and treat approach, there is limited evidence on the overall incidence of OIs at Amhara Regional State comprehensive specialized hospitals. In addition, previous studies have not considered rapid initiation of ART as an independent predictor of incidence of OIs.

This study was conducted in support of the Ethiopian HSTP-II goal of reducing HIV/AIDS and its complications by the end of 2025, as well as the targets of the Sustainable Development Goals (32). The results will help health professionals and clinicians by increasing existing knowledge, enabling prioritization of identified predictors, and identifying further measures for prevention and management of OIs. The study will provide input to program planners and decision-makers at various levels in the domain of HIV/AIDS care and support, and will also provide input for the implementation of regional and national programs. The findings will additionally serve as baseline data for further research.

Against this background, this study aimed to assess the incidence of opportunistic infections and the predictors of OI incidence among HIV-infected children receiving ART at Amhara Regional State comprehensive specialized hospitals, Ethiopia, in 2022.

## Methods and materials

### Study design, period, and setting

A multicenter institution-based retrospective follow-up study was conducted between May 17 and June 15, 2022. The study was conducted across seven Amhara Regional State comprehensive specialized hospitals (University of Gondar, Felege-Hiwot, Debre-Markos, Dessie, Woldia, Debre Birhan, and Debre Tabor).

The Amhara Region is one of the 11 regional states of Ethiopia and spans the northwestern, northeastern, and north-central parts of Ethiopia, with an estimated area of 159,173.66 square kilometers. It is administratively organized into 12 zones, three city administrations, and 183 woredas. According to the January 2022 Ethiopian Demographic and Health Survey report, the total population of the region is estimated at 30,848,988 (35). According to the annual performance report of the Amhara National Regional Health Bureau, the region has 858 health centers, 3,560 health posts, and 81 hospitals. These hospitals also have specialized units for ART care and related services (36).

Since 2005, the Amhara Regional State comprehensive specialized hospitals (CSHs) have been providing free ART services as part of the national AIDS control and prevention program. Seven of these hospitals were included in the study (the Tibebe-Ghion comprehensive specialized hospital was excluded due to an inadequate study population), and across these hospitals, 928 HIV-infected children were newly enrolled to receive ART from June 10, 2014, to February 28, 2022.

### Population selection and participation

The source population was all HIV-infected children aged <15 years and on ART follow-up in CSHs; the study population consisted of those newly enrolled for ART between June 10, 2014, and February 28, 2022. Newly HIV-infected children who had started receiving ART within this period were included in the study. Records with incomplete baseline information (CD4 count, hemoglobin level, weight, and height), unknown date of ART initiation, or unknown outcome status were excluded from the study.

### Sample size determination; sampling techniques and procedures

In order to determine the required sample size in relation to the first specific objective, we used a single-population proportion formula, considering an estimated incidence of opportunistic infections among HIV-infected children of 24.9%, which was taken from a study conducted at Debre Markos CSH 24.9% (12); this calculation yielded a sample size of 287.

For the second objective, the sample size was calculated on the basis of common significant predictor variables using Cox proportional hazard models, implemented in STATA version 16 (Table 1). This calculation yielded a sample size of 472 for the second objective, which was taken as the final required sample size for this study.

**Table 1:** Determination of estimated sample size required based on predictors of incidence of OIs among HIV-infected children receiving ART, using cox-model in STATA version 16.

Variable	AHR	Power	Probability of withdrawal	Probability of event	Sample size (*n*)
CPT					
Yes	-	80%	0.05	0.249	472
No	1.7				
ART adherence					
Good	-	80%	0.05	0.249	214
Poor/fair	2.2				
Anemia					
Yes	2.8	80%	0.05	0.249	119
No	-				

**Figure 1:**
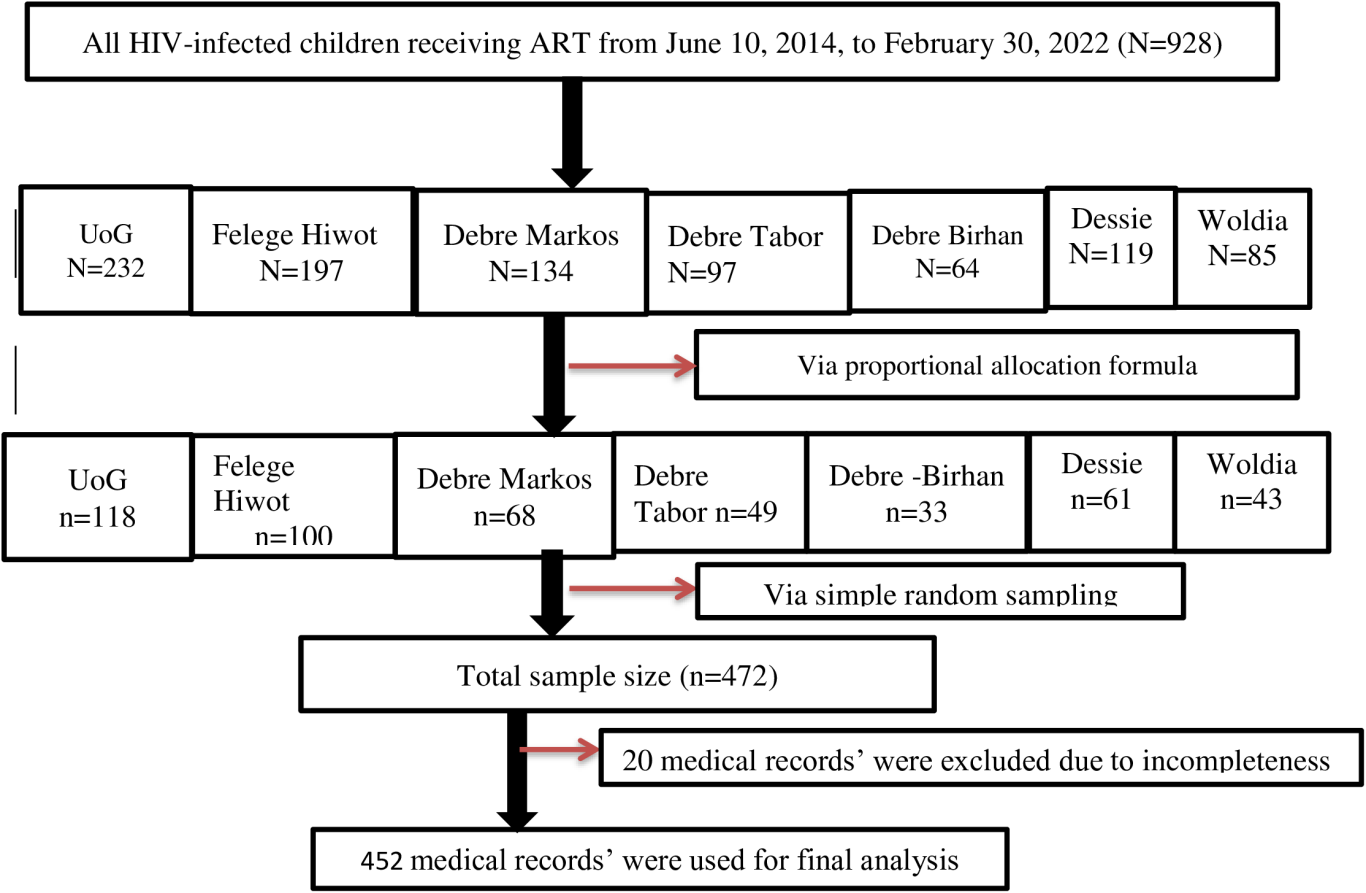
Schematic diagram of the sampling procedure for assessment of the incidence and predictors of OIs among HIV-infected children receiving ART at Amhara regional state comprehensive specialized hospitals in Ethiopia in 2022.

Samples were drawn proportionally from each of the seven CSHs, and records were selected using simple random techniques (Figure 1).

### Study variables and their measurements

The outcome variable was incidence of opportunistic infections among HIV-infected children receiving ART. The independent variables were: (1) sociodemographic characteristics (age, sex, residence, current HIV status of the parents, educational status of the caregiver, and marital status of the caregiver); (2) baseline clinical, nutritional, and laboratory characteristics (CD4 count, hemoglobin level, anthropometric indices, HIV disclosure status, functional and developmental status); (3) ART and medication-related characteristics: baseline ART regimen, presence of regimen change, level of ART adherence, receipt of TPT, receipt of CPT, ART side effects, and initiation of ART within seven days of diagnosis (4).

Opportunistic infections were considered for any type of infections with the clinical signs and symptoms of opportunistic disease occurring during the follow-up period (34). **An OI event** was deemed to have occurred when an HIV-infected child developed any OI after ART initiation during the follow-up period.

**Survival** was deemed to have occurred if the participant had no occurrence of any OI during the follow-up period.

**Censored**: Records of HIV-infected children (whether still living or not) were treated as censored if the child was transferred out to another health institution, was lost to follow-up, or was still on active ART follow-up but no evidence for any OI occurrence by the end of the study.

**Time to develop OI** was the time from ART initiation to the occurrence of an OI event during the follow-up period.

**Stunting** was recorded if the child had a height-for-age (HFA) or length-for-age (LFA) *Z*-score below −2 SD (34).

**Wasting** was recorded if weight-for-height (WFH) *Z*-score was less than −2 SD for children under 5 years old, or if body mass index (BMI)-for-age *Z*-score was less than −2 SD for children over 5 years old (34).

**Level of adherence to ART** was recorded as “good” in cases of compliance equal to or greater than 95% (or ≤ 3 missed doses per month); “fair” in cases of 85%–94% compliance (or between 4 and 8 missed doses per month); or “poor” in cases of less than 85% compliance (or ≥9 missed doses per month), as documented by ART health personnel in all cases (4).

**Rapid initiation of ART** was recorded in cases of initiation of ART care and support on the same day that HIV was confirmed or within 7 days (37).

**Anemia** (low hemoglobin level) was defined as a hemoglobin level of less than 10 mg/dl (12).

**CD4 count or percentage (%) below the threshold** was recorded based on thresholds specified by age category, as follows: CD4 cell count <1,500/mm^3^ or below 25% for children aged <12 months; CD4 cell count <750/mm^3^ or below 20% for children aged 12–35 months; CD4 cell count <350/mm^3^ or below 15% for children aged 36–59 months; and CD4 cell count <200/mm^3^ or below 15% for children aged age ≥60 months (34).

**Incidence of OIs** was calculated by dividing all newly occurring OI events by the total number of months of follow-up time across all patients.

### Data collection tool, procedure, and quality control

The data extraction tool was pretested on 5% of the sample size (22) 2 weeks before the actual data collection period at UoG CSH. Additionally, a 1-day practical training session on how to review ART follow-up and medical records, data collection methods, and the objective of the study was provided for the data collectors and supervisors. Data were collected using the KoBo Toolbox, which was prepared with relevant restrictions by trained nurses working in the selected hospitals. In addition to the data collector, supervisors and the principal investigator carefully monitored the entire data collection process and daily submission reports.

Data were collected from ART intake and follow-up forms and from children's charts using a data extraction tool adopted from the 2021 Ethiopian ART guidelines (34). Data were collected using smartphones by seven nurses with bachelor's degrees and three supervisors who were familiar with the ART follow-up process and had received basic ART training. The occurrence of OIs was ascertained during data extraction by reviewing health professionals’ reports on the children's charts.

### Data processing and analyses

The collected data were exported to STATA version 16 (MP) statistical software for management and analysis. The WHO anthro and anthroPlus software packages were used to generate anthropometric indices. Descriptive statistics (means, frequencies, and percentages) were computed and are presented in the tables and figures. In addition, a log-rank test was used to compare OI-free survival time for different levels of each categorical predictor variable. Multi-collinearity was tested by calculating the Variance Inflation Factor (VIF = 1.21) as a measure of the associations between predictor variables.

The assumptions of the Cox proportional hazard test were checked using Schoenfeld residuals and *via* a log-log plot all of the covariates (graphical check). The data fulfilled the assumptions, and the overall global model satisfied the proportional hazard assumption (global test, *P* = 0.9539).

**Figure 2:**
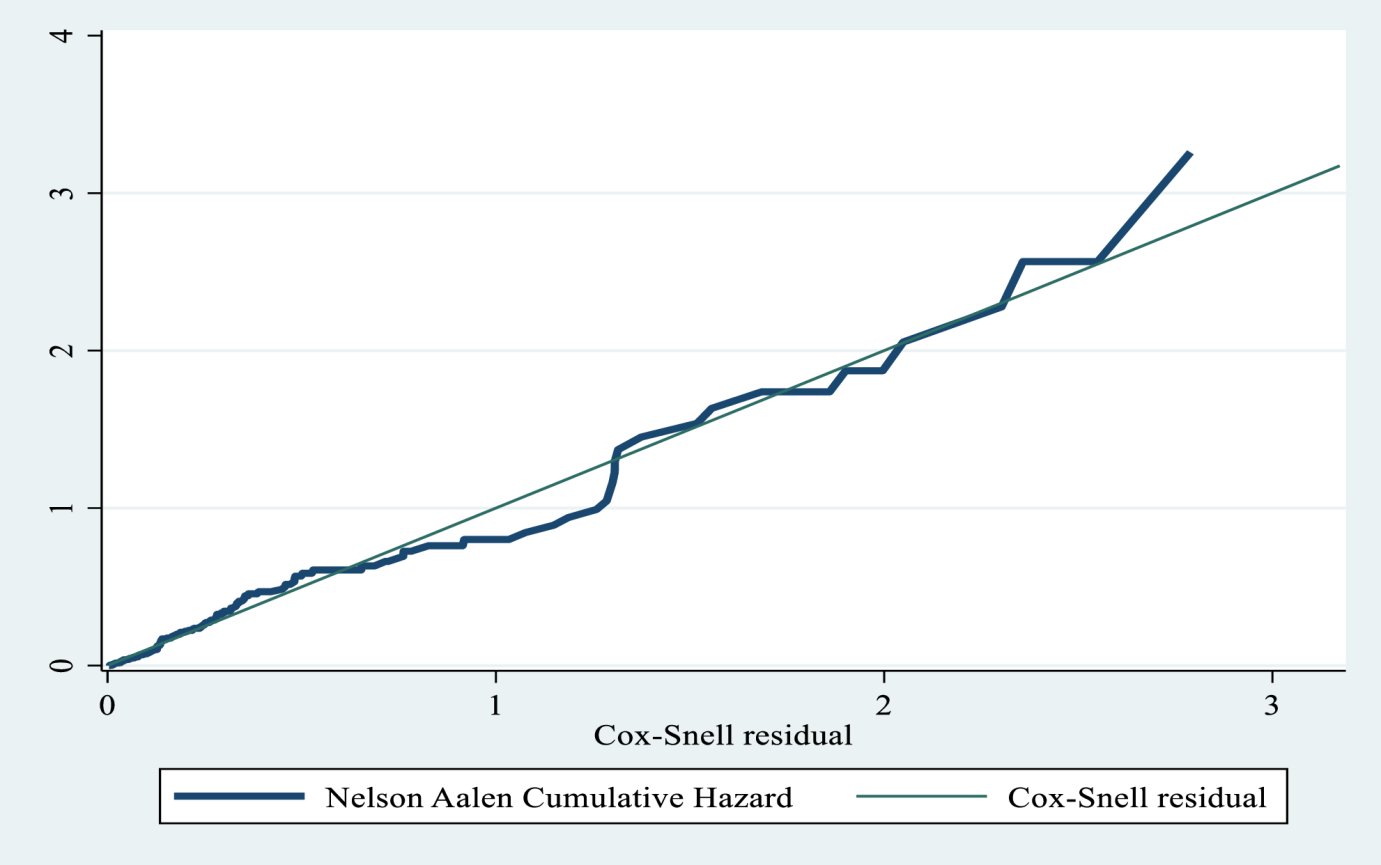
Goodness-of-fit test for the Cox proportional hazard regression model.

The Cox–Snell residual test was used to check the goodness of fit. A residual is a difference between an observed data point and a predicted or fitted value; as the graph in Figure 2 indicates, the Cox regression model fit with the Cox–Snell residual and the predicted hazard at 45°.

At this time, all assumptions were fulfilled. Shared frailty (theta) was estimated to be significantly different from zero (theta: 4.00e-19; 95% CI: 0; Prob ≥ chibar2 = 0.5); this shows that the distribution of unmeasured variables did not differ between Comprehensive Specialized Hospitals. A multivariable Cox proportional hazards model was used to identify predictors. A 95% confidence interval (CI) for adjusted hazard ratio (AHR) was estimated to determine the strength of associations. A *P*-value ≤0.05 was taken to indicate statistical significance for associations between predictors and incidence of OIs.

## Ethical considerations

Ethical clearance was obtained from the Institutional Review Board (IRB) of the University of Gondar on behalf of the Ethical Review Committee of the School of Nursing with ref. no. SN/1228/2014 on 03/05/2022 (G.C) and with ref. no. SN/1228/2014. A written permission letter was obtained from Amhara Public Health Institute for each hospital (ref. no. አሕጤኢ/ዋ/ዳ/03/1438). Finally, before the data were collected, a letter of permission was obtained from each of the respective CSH administrators to collect data from each ART clinic. At the time of data extraction, personal identifiers (names and contact numbers) were removed. The data were kept strictly confidential and used only for research purposes.

## Results

### Socio-demographic characteristics of HIV-infected children receiving ART

A total of 452 HIV-infected children receiving ART participated in the study; their medical records were complete in 95.8% of cases. The mean (±SD) age of participating children was 7.43 (±4.16) years. Twenty-nine percent of children were in the <5 years age group; 58.2% of the children were boys, and more than two-thirds (70.6%) were urban residents. In terms of parental characteristics, 54.2% were married, 33.85% had no formal education, and 70% were living (Table 2).

**Table 2:** Socio-demographic characteristics of HIV-infected children receiving ART at Amhara regional state comprehensive specialized hospitals, Ethiopia, 2022 (*n *= 452).

Characteristics	Frequency (*n*)	Percentage (%)
Age (years)		
<5	133	29.42
5–9	154	34.07
10–14	165	36.5
Sex		
Female	263	58.2
Male	189	41.8
Residence		
Rural	133	29.42
Urban	319	70.58
Current status of parents		
Both parents living	318	70.35
One parent living	117	25.88
Both parents deceased	17	3.76
Educational status of the caregiver		
No formal education	157	34.73
Primary	138	30.53
Secondary	88	19.47
Tertiary (college and above)	69	15.27
Marital status of the caregiver		
Married	245	54.2
Unmarried	100	22.12
Divorced	73	16.15
Widowed	34	7.52

### Baseline clinical, nutritional, and laboratory characteristics

The majority of children (78.54%) had CD4 cell counts above the threshold; in terms of functional status, 72.87% were working; and 71.11% had appropriate motor developmental status. In terms of nutritional status, approximately 16.37% were anemic; wasting and stunting were present in 30.75% and 48.89% of children, respectively (Table 3).

**Table 3:** Baseline clinical, nutritional, and laboratory test characteristics of HIV-infected children receiving ART at Amhara regional state comprehensive specialized hospitals, Ethiopia, 2022.

Characteristics	Frequency	Percentage (%)
CD4 cell count		
Above threshold	355	78.54
Below threshold	97	21.46
Child's disclosure status (*n* = 320)		
Yes	143	44.69
No	177	55.31
Anemia		
No	378	83.63
Yes	74	16.37
Functional status (*n* = 317)		
Working	231	72.87
Ambulatory	83	26.18
Bedridden	3	0.95
Developmental status (*n* = 135)		
Appropriate	96	71.11
Delayed	35	25.93
Regressed	4	2.96
Wasting		
No	313	69.25
Yes	139	30.75
Stunting		
No	231	51.11
Yes	221	48.89

### ART and other medication-related characteristics

The proportion of children exhibiting good adherence to ART and receiving TPT and CPT during the follow-up period were 70.58%, 64.38%, and 82.74%, respectively. In addition, only 37.83% of participants had initiated ART within 7 days after admission (Table 4).

**Table 4:** ART and medication-related characteristics of HIV-infected children receiving ART at Amhara regional state comprehensive specialized hospitals, Ethiopia, 2022 (*n* = 452).

Characteristics	Frequency	Percentage
ART drug adherence		
Good	308	68.14
Fair	74	16.37
Poor	70	15.49
TPT taken		
Yes	291	64.38
No	161	35.62
CPT taken		
Yes	374	82.74
No	78	17.26
ART side effects		
No	296	65.49
Yes	156	34.51
Presence of regimen change		
Yes	227	50.22
No	225	49.78
Initiated ART within 7 days		
Yes	168	37.17
No	284	62.83
Baseline ART drug regimen		
AZT − 3TC − NVP	99	21.90
AZT + 3TC + EFV	93	20.58
AZT + 3TC + LPV/r	21	4.65
ABC + 3TC + LPV/r	64	14.16
ABC + 3TC + DTG	60	13.27
ABC + 3TC + EFV	55	12.17
ABC + 3TC + NVP	25	5.53
TDF + 3TC + EFV	22	4.87
TDF + 3TC + DTG	13	2.88

### Incidence of opportunistic infections among HIV-infected children receiving ART

Across all 452 HIV-infected children receiving ART, follow-up periods ranged from 1 to 92 months, with a mean observation period of 36.8 months. The total observation time was 16,667.00 person-months (1,388.92 PYO).

New cases of opportunistic infection were observed in 120 participants (26.55%, 95% CI: 22.67, 30.88) within the follow-up period, and 73.45% were censored.

The overall incidence of OIs among HIV-infected children receiving ART across the seven Amhara Regional State CSHs was 8.64 (95% CI: 7.22, 10.33) per 100 PYO. The incidences of OIs in Woldia, University of Gondar, and Felege Hiwot CSHs were 12.86, 10.43, and 5.46 per 100 PYO, respectively (Table 5).

**Table 5:** Incidence of OIs by hospital among HIV-infected children receiving ART at Amhara regional state comprehensive specialized hospitals, Ethiopia, 2022 (*n* = 452).

Name of hospital	Number of patients	OI cases	Incidence (cases per 100 PYO)
Gondar CSH	113	32	10.43
Debre Tabor CSH	49	13	8.66
Felege Hiwot CSH	97	18	5.46
Debre Markos CSH	65	19	10.19
Woldia CSH	38	16	12.86
Dessie CSH	58	14	7.72
Debre Birhan CSH	32	8	7.25
Total	452	120	8.64

CSH, comprehensive specialized hospital; PYO, person-years of observation.

**Figure 3:**
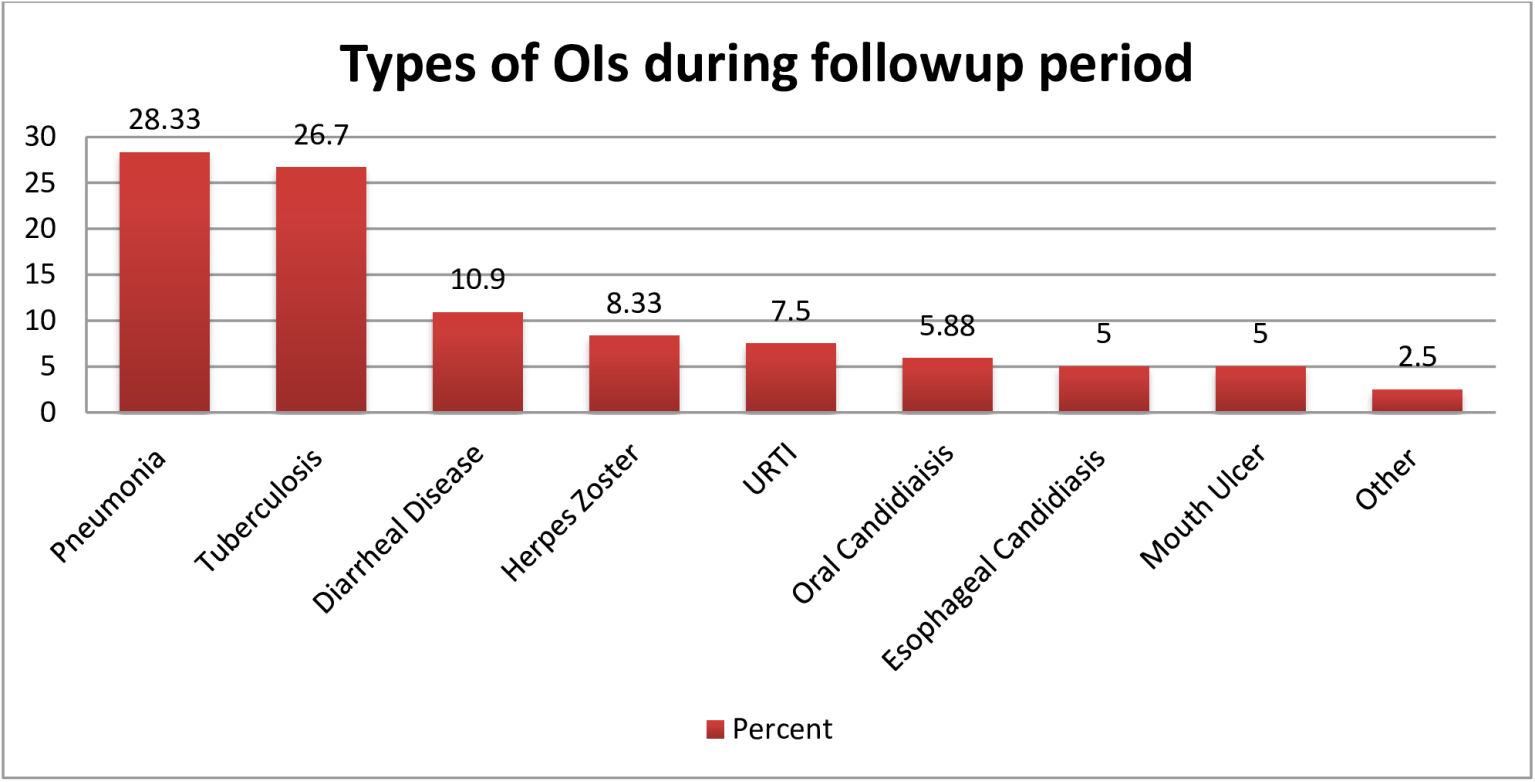
Common types of opportunistic infection observed among HIV-infected children receiving ART during the follow-up period at Amhara regional state comprehensive specialized hospitals, Ethiopia, 2022. Other = cryptococcal meningitis, Pneumocystis carinii pneumonia (PCP).

The mean survival time was 67.57 months (95% CI: 64.02, 71.12). During the follow-up period, pneumonia was the most commonly observed OI, affecting nearly 28.33% of children, followed by tuberculosis (26.7%), diarrheal disease (10.9%), herpes zoster (8.33%), and acute upper respiratory tract infections (7.5%) (Figure 3).

### Comparison of incidence of OIs among levels of categorical variables

The incidence of opportunistic infections per 100 PYO was found to be higher in children with anemia (24.75), those with a CD4 cell count below the threshold (18.71), those who reported never taking CPT (16.82) or TPT (15.76), and those who ever exhibited fair or poor adherence to ART (18.94) (Table 6).

**Table 6:** Incidence of opportunistic infections (cases per 100 PYO) among HIV-infected children receiving ART in Amhara regional state comprehensive specialized hospitals, 2022 (*n* = 452), stratified by variables investigated.

Variables	OI		Person-months of observation	OI IDR, cases per 100 PYO	*P*-value
	Event (*n* = 120)	Censored (*n* = 332)			
Marital status of the caregiver					
Married	54	191	9,315	6.96	0.011
Unmarried	24	76	3,483	8.27	
Divorced	30	43	2,498	14.41	
Widowed	12	22	1,371	10.5	
Initiated ART within 7 days					
Yes	22	146	5,721	4.61	0.0002
No	98	186	10,946	10.74	
CD4 cell count					
Above threshold	74	281	13,716	6.47	<0.001
Below threshold	46	51	2,951	18.71	
Anemia					
No	69	309	14,194.00	5.8	<0.001
Yes	51	23	2,473	24.75	
Duration of ART					
<6 months	43	34	2,522	14.27	<0.001
≥6 months	77	298	14,145	7.64	
Adherence to ART					
Good	51	261	12,296	4.98	<0.001
Fair or poor	69	71	4,371	18.94	
Ever taking tuberculosis preventive therapy					
Yes	54	237	11,641	5.57	<0.001
No	66	95	4,439.73	15.76	
Ever taking cotrimoxazole preventive therapy					
Yes	86	288	14,242	7.25	<0.001
No	34	44	2,425.00	16.82	
Wasting					
No	76	237	12,008.00	7.59	0.0431
Yes	44	95	4,659.00	11.33	

IDR, incidence density rate.

The incidences of OIs within follow-up periods of less than 6 months, between 6 and 12 months, between 12 and 36 months, between 36 and 72 months, and between 72 and 92 months were 14.27, 7.51, 7.08, 9.22, and 1.97 per 100 PYO, respectively. In this study, the incidence of OIs in the first 6 months of follow-up was high, at 14.27 per 100 PYO.

### Kaplan–Meier failure function or probability of OI

**Figure 4:**
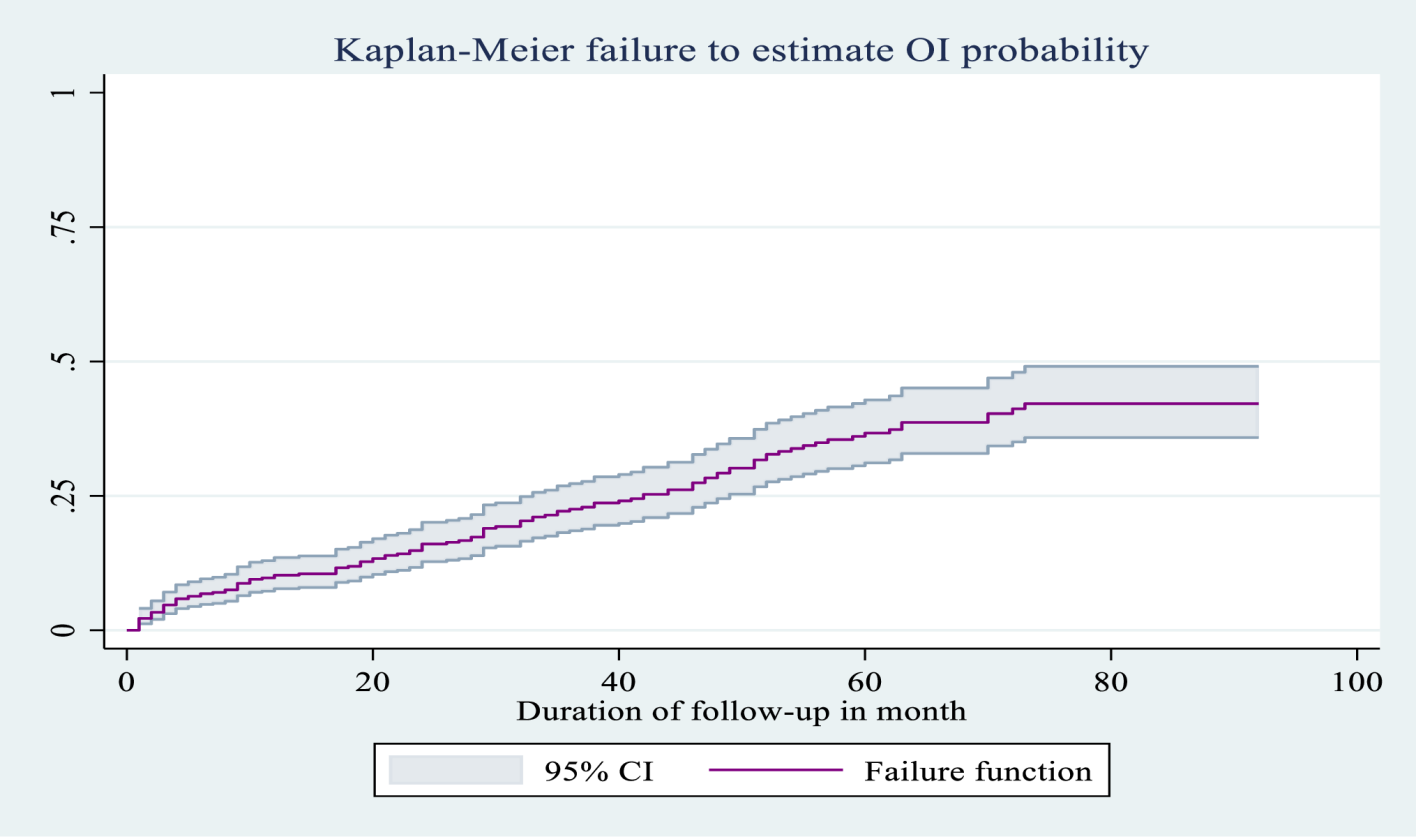
Overall Kaplan–Meier failure as an estimate of the probability of occurrence of OI (with 95% confidence interval) among HIV-infected children receiving ART at Amhara regional state comprehensive specialized hospitals, Ethiopia, 2022.

The probability of an OI occurring during the entire follow-up period was 0.424 (95% CI: 0.358, 0.497) by the end of follow-up; 0.064 (95% CI: 0.045, 0.0.091) after 6 months; 0.098 (95% CI: 0.0.074, 0.131) after 1 year; 0.218 (95% CI: 0.179, 0.264) after 3 years; 0.398 (95% CI: 0.339, 0.464) after 5 years; and 0.424 (95% CI: 0.358, 0.497) after 7 years (Figure 4).

### Comparison of probability of OI-free survival among levels of categorical variables

**Figure 5:**
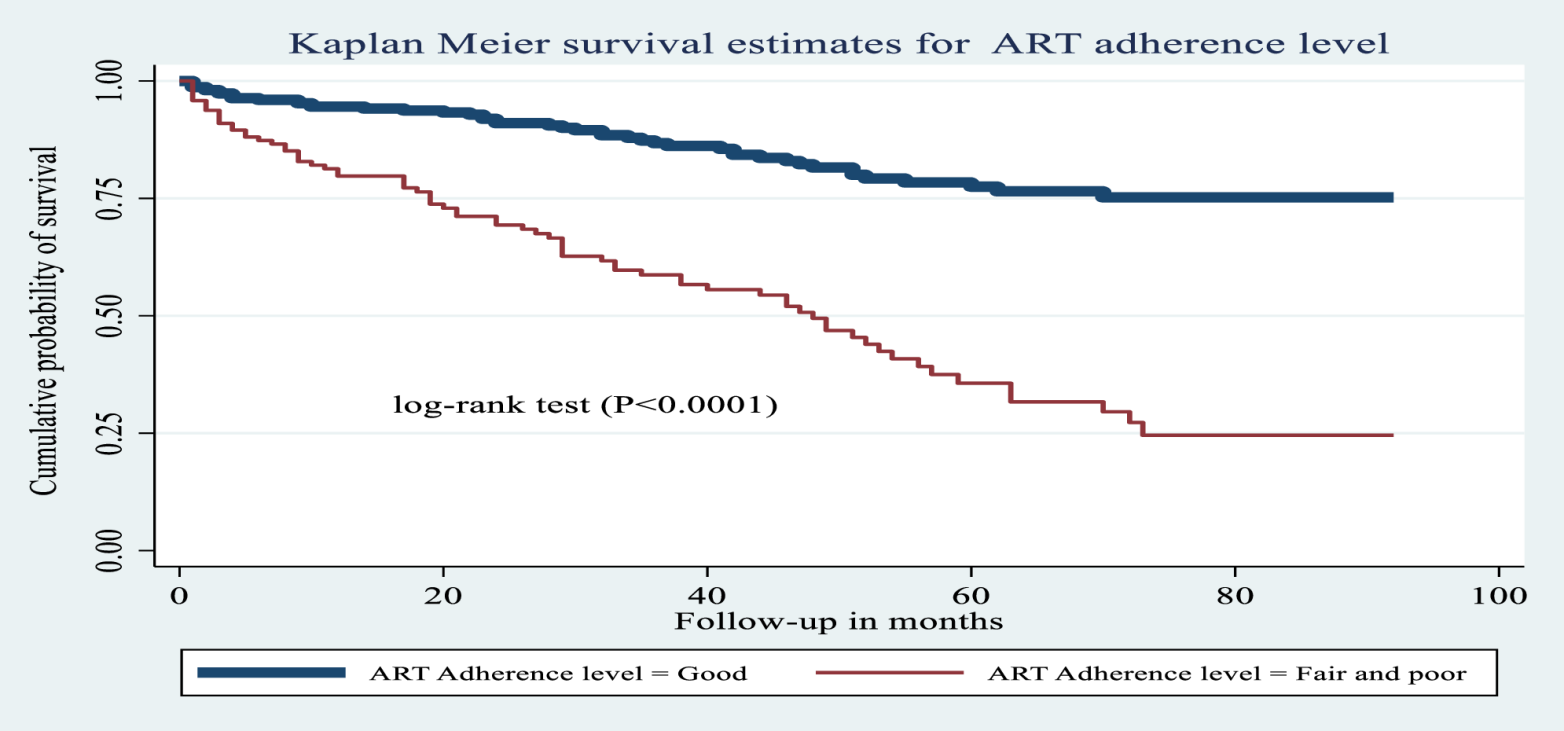
Kaplan–Meier estimates of probability of OI-free survival by ART adherence level among HIV-infected children receiving ART at Amhara regional state comprehensive specialized hospitals, Ethiopia, 2022.

**Figure 6:**
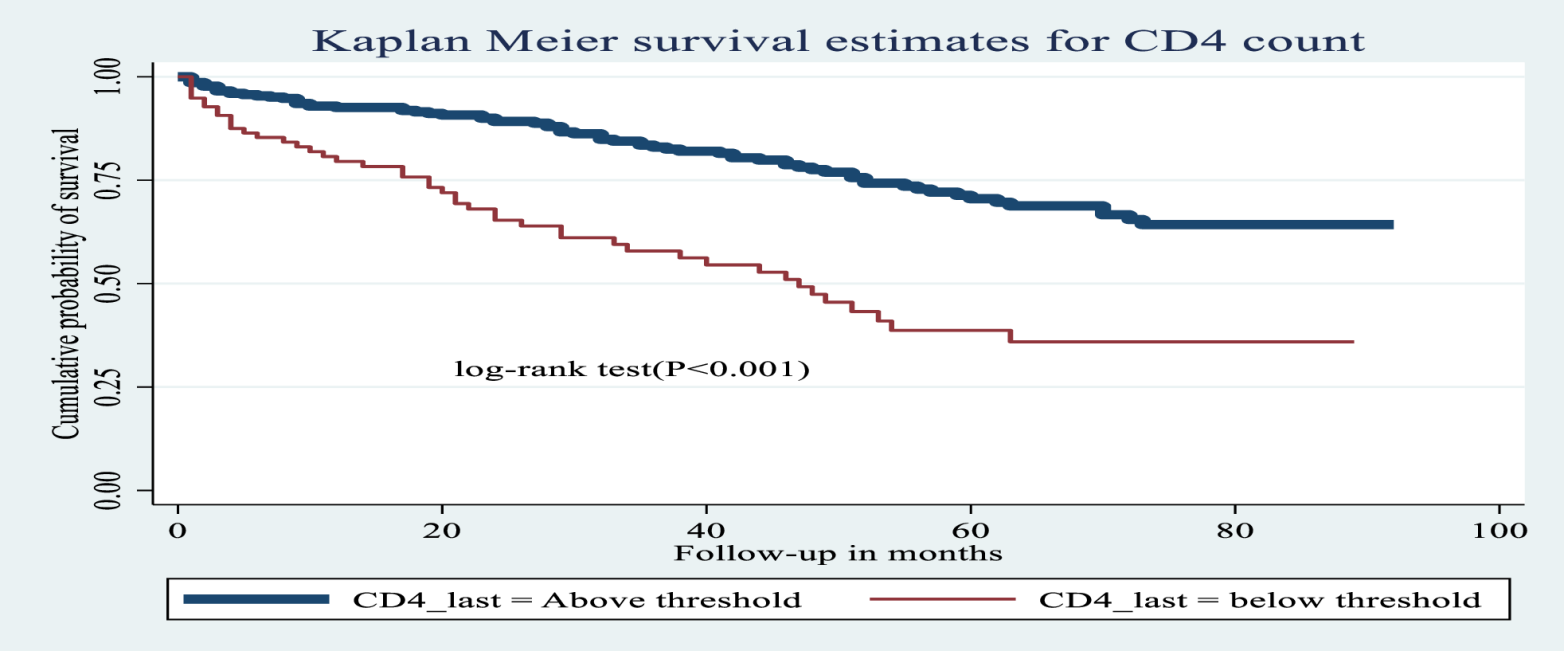
Kaplan–Meier estimates of probability of OI-free survival by CD4 cell count (above or below threshold) among HIV-infected children receiving ART at Amhara regional state comprehensive specialized hospitals, Ethiopia, 2022.

**Figure 7:**
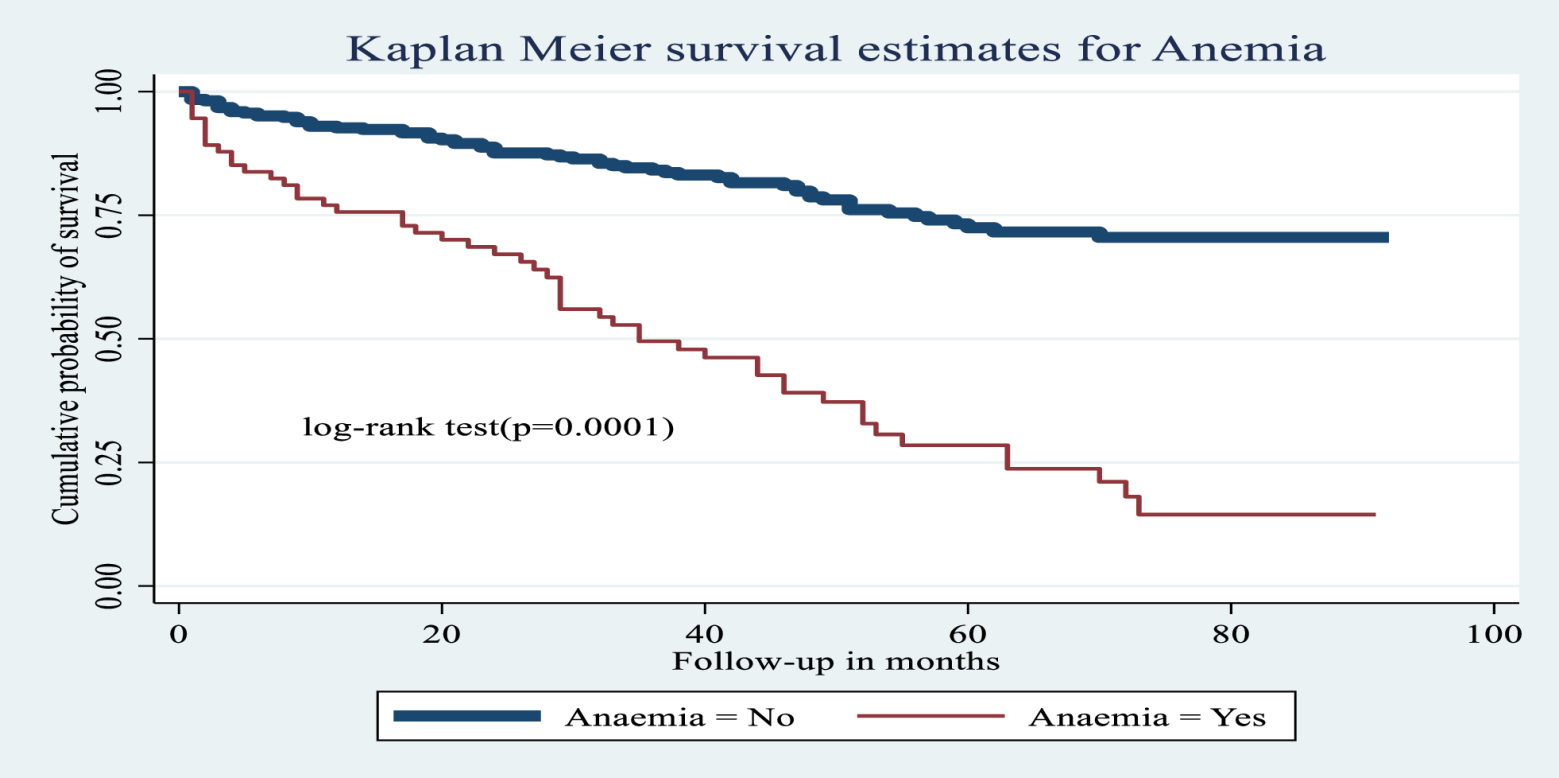
Kaplan–Meier estimates of probability of OI-free survival with and without anemia among HIV-infected children receiving ART at Amhara regional state comprehensive specialized hospitals, Ethiopia, 2022.

**Figure 8:**
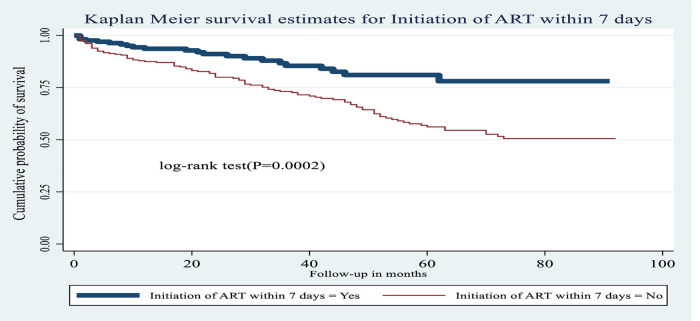
Kaplan–Meier estimates of probability of OI-free survival by ART initiation time among HIV-infected children receiving ART at Amhara regional state comprehensive specialized hospitals, Ethiopia, 2022.

Children who ever exhibited poor or fair adherence to ART during the follow-up period had a lower probability of OI-free survival compared to those who exhibited good adherence to ART (log-rank test, *χ*^2^ = 66.91, *P* < 0.0001). Comparisons of OI-free survival probability among levels of this and other categorical variables are shown in Figures 5–8.

### Predictors of incidence of OIs among HIV-infected children receiving ART at Amhara regional state CSHs

In the multivariable Cox regression analysis, only CD4 cell count, anemia status, ART adherence level, ever taking TPT, and initiation of ART within 7 days of admission showed statistically significant associations with incidence of OIs among HIV-infected children receiving ART.

CD4 cell count showed a statistical association with incidence of OIs: specifically, the hazard of developing OIs among children presenting with a CD4 cell count below the threshold was 2.34 times greater [AHR: 2.34 (95% CI: 1.45, 3.76)] than among children with a CD4 count above the threshold.

The hazard of developing OIs among children with anemia was 1.68 times greater [AHR: 1.68 (95% CI: 1.06, 2.67)] than among non-anemic children.

Adherence to ART was also found to be statistically significant factor for incidence of OIs: specifically, HIV-infected children receiving ART who exhibited fair or poor ART adherence were 2.31 times more likely [AHR: 2.31 (95% CI: 1.47, 3.63)] to develop OIs than those children who exhibited good ART adherence.

The risk of developing OIs among HIV-infected children receiving ART who never took TPT was 1.95 times greater [AHR: 1.95 (95% CI: 1.27, 2.99)] than that of those who did take TPT during the follow-up period.

Lastly, the risk of developing OIs among HIV-infected children who did not initiate ART within 7 days of admission was 1.82 times greater [AHR: 1.82 (95% CI: 1.12, 2.96)] than among those who had initiated ART within 7 days, or immediately after diagnosis. Predictors of the incidence of OIs among HIV-infected children receiving ART are listed in Table 7.

**Table 7:** Bi-variable and multivariable Cox proportional hazard analysis of predictors of incidence of OIs among HIV-infected children receiving ART at Amhara regional state comprehensive specialized hospitals, Ethiopia, 2022 (*n* = 452).

Predictors	OI status		CHR (95% CI)	AHR (CI)
	Event (*n* = 120)	Censored (*n* = 332)		
Age in years				
<5 years	44	89	1.61 (1.04, 2.48)	1.18 (0.68, 2.06)
5–9	38	116	0.95 (0.61, 1.49)	1.14 (0.69, 1.87)
10–14	38	127	**1**	**1**
Status of parents				
Both alive	71	247	1	1
One or both deceased	49	85	1.88 (1.31, 2.71)	1.11 (0.71, 1.73)
Marital status				
Married	54	191	1	1
Never married	24	76	1.20 (0.74, 1.94)	1.42 (0.85, 2.36)
Divorced	30	43	2.08 (1.32, 3.24)	1.29 (0.76, 2.22)
Widowed	12	22	1.51 (0.81, 2.82)	1.09 (0.54, 2.23)
Educational status				
No formal education	46	111	2.02 (1.07, 3.82)	1.44 (0.73, 2.84)
Primary	39	99	1.74 (0.92, 3.34)	1.52 (0.77, 2.99)
Secondary	23	65	1.71 (0.85, 3.44)	1.15 (0.55, 2.44)
Tertiary	12	57	1	1
Initiated antiretroviral therapy within 7 days				
Yes	22	146	1	1
No	98	186	2.34 (1.47, 3.73)	1.82 (1.12, 2.96)*
CD4 count relative to threshold				
Above	74	281	1	1
Below	46	51	2.80 (1.93, 4.05)	2.34 (1.45, 3.76)***
Anemia status				
No	69	309	1	1
Yes	51	23	4.20 (2.92, 6.04)	1.68 (1.06, 2.67)*
Wasting				
No	76	237	1	1
Yes	44	95	1.46 (1.01, 2.12)	1.11 (0.70, 1.74)
Stunting				
No	58	173	1	1
Yes	62	159	1.36 (0.94, 1.94)	1.11 (0.74, 1.68)
ART adherence level				
Good	51	261	1	1
Fair or poor	69	71	4.11 (2.84, 5.94)	2.31 (1.47, 3.63)***
Ever taking tuberculosis preventive therapy				
Yes	54	237	1	1
No	66	95	2.78 (1.94, 3.99)	1.95 (1.27, 2.99)**
Ever taking cotrimoxazole preventive therapy				
Yes	86	288	1	1
No	34	44	2.28 (1.53, 3.41)	1.32 (0.79, 2.19)
ART side effects				
No	70	226	1	1
Yes	50	106	1.24 (0.86, 1.79)	1.49 (0.99, 2.25)
Presence of regimen change				
Yes	62	165	0.79 (0.56, 1.14)	1.09 (0.71, 1.66)
No	58	167	1	1
Initial drug regimen type				
Non-AZT	65	174	1	1
Including AZT	55	158	0.75 (0.52, 1.07)	0.78 (0.52, 1.17)

CHR, crude hazard ratio; 1, reference.

* Significant at <0.05.

** Significant at <0.01.

*** Significant at <0.001.

## Discussion

In this study, we planned to assess the incidence of OIs and predictors of their occurrence among HIV-infected children receiving ART in Amhara Regional State CSHs in 2022; we found that the overall incidence of OIs among HIV-infected children receiving ART within the study area was 8.64 (95% CI: 7.22, 10.33) per 100 PYO. This finding was consistent with that of a study conducted in Debre Markos CSH, Ethiopia (9.7 per 100 PYO) (8). However, this was a higher rate than observed in a study conducted in Northwest Ethiopia at the UoG and Debre Tabor CSHs (5.53 per 100 PYO) (12). These discrepancies in findings resulted from outcome ascertainment, in that our study considered all types of OIs, while the aforementioned studies focused only on advanced OIs. ART on the same day of HIV status confirmation without intensive counseling and adequate preparation may lead to poor adherence in the early phase of ART intuition which increase the incidence of OIs (37, 38).

Additionally, the incidence of OIs observed in this study was higher than that of studies conducted in the United States of America (4.99 per 100 PYO) (39), Latin America (1.1 per 100 PYO) (13), and Brazil (2.63 per 100 PYO) (14). This difference is due to developed countries and middle-income countries providing better healthcare services and facilities to prevent the occurrence of OIs in comparison to resource-limited countries like Ethiopia. Additionally, dissimilarities could be due to low levels of education (in this study, two-thirds of the participants’ caregivers had no formal education or a primary education only), poverty, the burden of communicable diseases due to poor hygiene and sanitation, overcrowded living conditions, and malnutrition, which are common problems in developing countries (5) that could further exacerbate immunosuppression and co-morbidity with other diseases.

In contrast to this study, another study conducted in Latin America found a higher incidence of OIs among HIV-infected children (23.5 per PYO) (11). This discrepancy results from the nature of the study designs: the Latin America study was a prospective cohort study in which data were gathered using unbiased techniques [observation and clinical evaluation: physical examinations, medical history, and advanced laboratory investigations (viral load test)], as well as regular follow-ups that could increase the detection of new OIs. Another possible reason for the discrepancy could be the timing of the study period, given the recent accessibility of new ART drugs with reduced side effects, such dolutegravir. In the current study, 34.51% of the participants reported experiencing side effects from ART (11, 34).

Many predictors showed statistically significant associations with the incidence of OIs among HIV-infected children receiving ART. In this regard, children who presented with a CD4 cell count below the threshold were 2.34 times more likely to develop OIs [AHR: 2.34 (95% CI: 1.45, 3.76)] than those children with a CD4 cell count above the threshold. The finding is supported by studies conducted in Latin America (11), India (21), the USA (19), and Northern Ethiopia (40), and at Debre Markos CSH (8). The reason is that CD4 cells play an important role in mounting an immune response to infections. These cells release cytokines that lead to the activation of antigen-presenting cells, phagocytic cells, natural killer cells, and cytotoxic T cells. A decrease in CD4 cell count thus leads to a decline in both humoral and cell-mediated immunity (41). This results in the depletion of CD4 cells and immunodeficiency, and the likelihood of OIs increases (21, 42).

The risk of developing OIs was 1.68 times greater among children with anemia [AHR: 1.68 (95% CI: 1.06, 2.67)] as compared to non-anemic children. This finding is supported by various studies conducted at Debre Tabor and University of Gondar CSHs (12), in the Metekel zone (43), and in Addis Ababa (44), Northern Ethiopia (40), Adama (29), Nigeria (45), Uganda (23), Tanzania (25) and South Africa (24, 46). The occurrence of anemia at the start of a chronic disease is caused by baseline pro-inflammatory cytokine upregulation. Evidence suggests that the dysregulated iron axis in HIV-related anemia may provide highly favorable intracellular macrophage conditions for microorganisms like TB bacilli, and subsequently results in the development of OIs (24, 47). Another clinical reason could be that CD4 cell count is significantly associated with the likelihood of anemia; this is explained by an increased viral burden, which may cause anemia through increased cytokine-mediated myelosuppression and a higher burden of OIs (48). Anemia in turn exerts an effect on O_2_ intake capacity, which leads to a synergistic effect with OIs, worsening the prognosis of the disease process and potentially ending in death (48).

HIV-infected children receiving ART who exhibited fair or poor ART adherence were 2.31 times more likely [AHR: 2.31 (95% CI: 1.47, 3.63)] to develop OIs as compared to those children who exhibited good ART adherence. This result is consistent with studies conducted at Debre Markos CSH (8), at Debre Tabor and University of Gondar (12), at Bahir Dar Public Hospitals (49), in Brazil (22), and in India (50). A possible reason that poor or fair adherence to a regime of HIV medications directly increases viral replication to its maximum, compromising health and quality of life; thus, the risk of developing OIs is maximized, and the risk of drug resistance is increased (34, 37, 38).

The risk of developing OIs among children who never took TPT was 1.95 times higher [AHR: 1.95 (95% CI: 1.27, 2.99)] than among those who took TPT during the follow-up period. This finding is comparable with findings reported in Nigeria (51), at UoG (52), in Northwest Ethiopia Gondar (53), in Adama (29), in Addis Ababa (54), at SNNP Region Hospitals (55), and at Arba Minch Hospital (56). This implies that TPT plays a role in decreasing mycobacterium load and reducing the progression of latent bacilli to active TB (57).

The risk of developing OIs among HIV-infected children receiving ART was 1.82 times higher among those who had not initiated ART within 7 days of admission or enrolment [AHR: 1.82 (95% CI: 1.12, 2.96)] than among those who had initiated ART within this time frame. Recently, the WHO has strongly recommended initiating ART on the same day that HIV is confirmed, or within 7 days, after ensuring the patient's willingness and readiness to start ART immediately (37). If children do not start ART quickly, the incidence of OIs is likely to be increased. Early ART commencement improves drug uptake, HIV care retention, and viral suppression (58). By 2025, 95% of patients undergoing ART will have achieved viral suppression, which will play a role in lowering the incidence of OIs. This might be achieved through rapid ART commencement (59).

Despite the interesting research topic and strong methodological considerations throughout the course of our study, the retrospective nature of data collection obliged us to rely on existing and previously recorded information on the type of OIs that occurred during follow-up, which could mean that important variables were missed. Likewise, the use of subjective measures (given the limited capacity to establish definitive diagnoses of OIs) means that asymptomatic OIs could surely have been missed, in turn resulting in an underestimate for the study's outcome of interest. Additionally, considering the use of children's charts as data sources, non-registered variables that could have influenced the occurrence of OIs (income, caregiver's occupational status, family size, and viral load) were not investigated.

## Conclusion

In this study, a high incidence of OIs was observed during a specified follow-up period among HIV-infected children receiving ART at Amhara Regional State CSHs. Having a CD4 cell count below the threshold, ever exhibiting only fair or poor adherence to ART, co-morbidity of anemia, ever taking TPT, and not having initiated ART within 7 days were significant predictors of an increased incidence of OIs among this group.

## Recommendation

Caregivers are recommended to bring their children for early screening, and to engage with follow-up care to the greatest extent possible. CSHs should closely monitor children who present with a baseline CD4 cell count below the threshold, or with a low hemoglobin level or anemia, and should place more emphasis on working with the caregivers of HIV-infected children in order to achieve good ART adherence during the follow-up period. Ideally, ART should be initiated immediately or within 7 days of the HIV diagnosis being confirmed. Further prospective cohort studies should be conducted, incorporating important predictors of OIs like income status, occupational status of the caregiver, family size, and viral load; further qualitative research on ART drug adherence level should also be conducted.

## Data Availability

The original contributions presented in the study are included in the article, further inquiries can be directed to the corresponding author.
